# DnaG Primase—A Target for the Development of Novel Antibacterial Agents

**DOI:** 10.3390/antibiotics7030072

**Published:** 2018-08-13

**Authors:** Stefan Ilic, Shira Cohen, Meenakshi Singh, Benjamin Tam, Adi Dayan, Barak Akabayov

**Affiliations:** Department of Chemistry, Ben-Gurion University of the Negev, Beer-Sheva 8410501, Israel; ilic@post.bgu.ac.il (S.I.); shiracoh@post.bgu.ac.il (S.C.); meenaksh@post.bgu.ac.il (M.S.); tamb@post.bgu.ac.il (B.T.) adiday@post.bgu.ac.il (A.D.)

**Keywords:** DNA replication, DnaG primase, bacterial inhibitors, antibacterial agents, antibiotics

## Abstract

The bacterial primase—an essential component in the replisome—is a promising but underexploited target for novel antibiotic drugs. Bacterial primases have a markedly different structure than the human primase. Inhibition of primase activity is expected to selectively halt bacterial DNA replication. Evidence is growing that halting DNA replication has a bacteriocidal effect. Therefore, inhibitors of DNA primase could provide antibiotic agents. Compounds that inhibit bacterial DnaG primase have been developed using different approaches. In this paper, we provide an overview of the current literature on DNA primases as novel drug targets and the methods used to find their inhibitors. Although few inhibitors have been identified, there are still challenges to develop inhibitors that can efficiently halt DNA replication and may be applied in a clinical setting.

## 1. Introduction

The complex process of identifying antibacterial compounds begins with the selection of potential targets, which must be essential, selective over human homologues, susceptible to drugs, and have a low propensity to develop a rapid resistance [1]. Even though bacteria possesses approximately 200 essential gene products, only a limited number of these have been exploited as drug targets [2]. DNA replication, which qualifies as a novel drug target, is performed by the replisome. A replisome is a multi-enzyme complex that synthesizes DNA continuously on its leading strand and discontinuously on its lagging strand [3,4]. Within a replisome of every living cell, DNA primase is an essential component that synthesizes short RNA primers that are used by DNA polymerase to form the “Okazaki fragments” on the lagging DNA strand [5]. After primer extension, the RNA part is removed by RNase H and the gap is filled with DNA by DNA polymerase I. 

The inhibition of primase is expected to halt DNA replication and, as a result, cell proliferation. The potential of making DNA primase a clinical target for novel antibiotics is high, but has not resulted in an increase of the repertoire of clinical candidates that could be especially beneficial against drug-resistant bacteria. This review provides a current literature survey on DnaG primase and the properties that make this enzyme an attractive bacterial target. The challenges in finding ways to inhibit primase are discussed along with the current drug discovery tools that have been used to develop novel and effective inhibitors. Examples of currently available primase inhibitors are provided as well. 

## 2. The Bacterial Replisome as a Multiple-Drug Target

**Figure 1:**
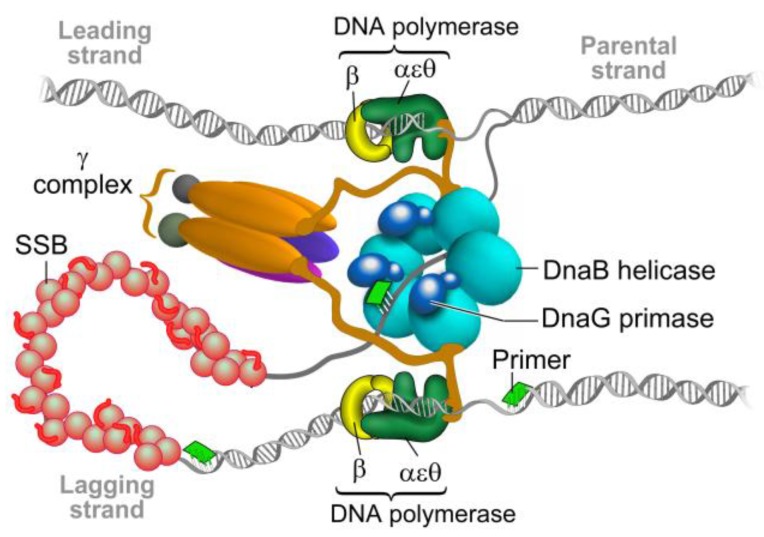
The bacterial replisome. The helicase DnaB unwinds double-stranded DNA and exposes the two individual strands. One strand is copied continuously (leading) and the other discontinuously (lagging) by two DNA polymerase III complexes. Each DNA polymerase is attached to the DNA by the β clamp to allow DNA synthesis to proceed. On the lagging strand, the DNA polymerase uses short RNA primers synthesized by primase DnaG to form the “Okazaki fragments”. The RNA primers on the lagging strand are degraded and the gaps are filled and ligated by further enzymatic activities in the replication fork, which forms a single continuous DNA strand. Modified from Reference [9].

Replication of the chromosome is a central event in the cell cycle of every bacterium. It is performed by the replisome, which is a multi-enzyme complex that synthesizes DNA continuously at the leading strand and discontinuously at the lagging strand ([6], Figure 1). A bacterial replisome is composed of multiple subunits and the activities of individual components are highly coordinated to achieve efficient and accurate DNA replication [3]. The components of bacterial replisomes from model systems such as *Escherichia coli* and *Bacillus subtilis* have been characterized extensively, which revealed the molecular mechanisms at the DNA replication apparatus [7,8]. Understanding the basic mechanisms that regulate the replication of DNA—which are largely unexplored in many pathogenic bacteria such as mycobacteria including *Mycobacterium tuberculosis*—are critical for the development of new therapeutic approaches to control bacterial proliferation [1].

Targeting new biochemical pathways—such as DNA replication—is directed against resistant strains of pathogenic bacteria. Some of those biochemical pathways present a different setting in humans, which provides high selectivity. Inhibiting any of the essential enzymes associated with DNA replication should affect bacterial growth. Several anti-bacterial agents that target DNA replication proteins such as PolIII (including the core α subunit, β clamp, and the clamp loader complex), DnaB helicase, and a single-stranded binding protein (SSB) are currently under development (for review *see* [10]). Although DNA replication machinery is a promising multi-drug target, only quinolones that target TopoII (Gyrase), which is an enzyme downstream from the DNA replication fork that relieves strain of dsDNA during replication by active formation of negative supercoiling. These factors have found their way to the clinic [11]. Quinolones, however, don’t inhibit TopoII but rather convert it into a toxic form that causes fragmentation of the bacterial genome [12,13,14,15,16,17]. 

### Examples for Unique Potential DNA Replication Targets

Inhibition of DNA replication has been shown to provide an effective antibacterial activity. The cyclic peptide griselimycin that targets DnaN (the β clamp subunit of DNA polymerase III) halts DNA replication of *M. tuberculosis* and, as a result, kills bacteria [18]. Another example for the potential of DNA replication proteins to become a useful target in antibiotic discovery is the histidinol phosphatase (PHP)-exonuclease domain of DnaE from *M. tuberculosis*. A recent crystal structure of DnaE1 from *M. tuberculosis* reveals that the PHP-domain has some unique structural features, which make it an attractive target for novel anti-mycobacterial drugs [19].

Other examples of antimicrobial agents that affect bacterial replisomes include aminocoumarins and quinolones that target DNA gyrase and DNA topoisomerase IV to halt growth of *Staphylococcus aureus* [20]. Aminocoumarins compete with ATP on binding to the Gyrase B subunit while quinolones stabilize the DNA-cleavage complex [16,21]. Similarly, inhibitors of metabolic pathways of purine nucleotide synthesis have been shown to affect bacterial growth by inhibiting DNA replication [22]. There are several more examples. However, the full potential of DNA replisome as a multiple drug target is far from being achieved. 

## 3. Structural Features of DnaG Primase: Opportunities for Drug Targeting

**Figure 2:**
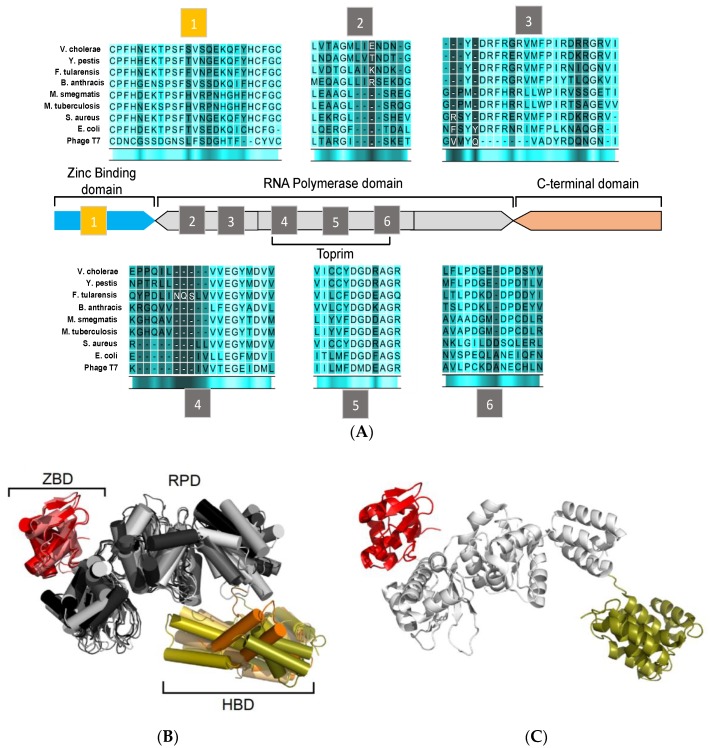
Sequence and structural homology of DnaG-like primases. (**A**) Domain organization and arrangement of motif sequences of prokaryotic DNA primases. (**B**) Structural alignment of DnaG primase domains: zinc-binding domain (ZBD) of *A. aeolicus* (PDB ID 2AU3), and *B. stearothermophilus* (PDB ID 1D0Q). RNA polymerase domain (RPD) of *A. aeolicus* (PDB ID 2AU3), *B. subtilis* (PDB ID 5GUJ), *E. coli* (PDB ID 1DD9), *M. tuberculosis* (PDB ID 5W33), *Pseudomonas aeruginosa* (PDB ID 5VAZ), and *S. aureus* (PDB ID 4E2K). C-terminal domain of *B. stearothermophilus* (PDB ID 1Z8S), *E. coli* (PDB ID 2HAJ), *H. pylori* (PDB ID 4EHS), *S. aureus* (PDB ID 2LZN), and *V. cholerae* (PDB ID 4IM9). The ZBD is colored in shades of red. The RPD is colored in shades of gray and the C-terminal domain is colored in shades of orange. (**C**) Representative model of bacterial DnaG primase consisting of ZBD (colored red) and RPD (colored white) of *A. aeolicus* (PDB ID 2AU3) and the C-terminal domain (colored deep olive) of *S. aureus* (PDB ID 2LZN). The figure was created using the PyMOL (http://www.pymol.org) and CLC Sequence viewer 6.

DNA primase is a central component in the core replisome of every living cell. This enzyme synthesizes short RNA primers of approximately 10 nucleotides long, which are delivered to the DNA polymerase for extension to form Okazaki fragments on the lagging DNA strand. Prokaryotic DnaG primases are similar both in sequence and structure (Figure 2). 

**Figure 3:**
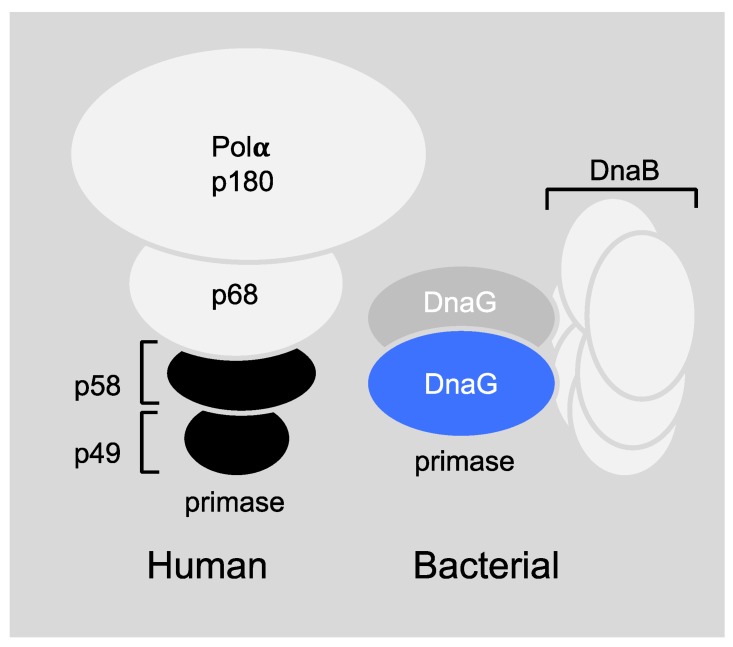
Schematic models of prokaryotic primase vs. eukaryotic primase. (**Left**) The DNA polymerase α–primase complex from human consists of four subunits. The p180 subunit is pol α, p58 and p49 comprise primase and p68 is the fourth, tightly bound subunit. (**Right**) DnaG primase interacts with hexameric DnaB helicase, however, the exact stoichiometry of this interaction is not known.

The differences in the setting of mammalian and prokaryotic priming during DNA replication are profound (Figure 3) and make the bacterial primase an ideal target for drug design. More specifically, the human primase consists of four subunits (Figure 3, left), the primase core P49, the primase accessory protein P58, DNA polymerase β, P180, and the accessory protein P68 [23]. However, the bacterial DnaG primase usually works in accordance with the DnaB helicase hexameric ring (Figure 3, right) even though the stoichiometry of this interaction is not fully known to date (for more information see Section 6.1). In addition, the sequence homology between the mammalian and bacterial primase is very low [5]. Bacterial primase contains an active site for binding nucleotides and a DNA binding module, which makes it “druggable.” All these features make primase an excellent therapeutic target, but even though extensive efforts to find inhibitors for DnaG primase have been made over the years, no clinical candidate has been developed. 

Bacterial DnaG primases are composed of three main domains (Figure 2): The N-terminal zinc binding domain (ZBD), the RNA polymerase domain (RPD), and the C-terminal helicase binding domain (HBD). While crystal structures of the DnaG domains have been reported, a full DnaG structure remains undisclosed. 

The ZBD contains a four-stranded, antiparallel β sheet and a highly conserved three cysteine and one histidine residues mostly located on loop regions. These residues coordinate the zinc ion and form a typical zinc-ribbon structure, which is also commonly known as the zinc finger motif. The zinc finger motif mediates DNA binding where the zinc ion plays a role in DNA sensing and stabilization of the motif [24,25]. The structure of ZBD is still poorly characterized since the information can only be obtained from two available structures of DnaG-ZBD from *B. stearothermophilus* (PDB ID 1D0Q [26]) and *Aquifex aeolicus* (PDB ID 2AU3 [27]) (Figure 2B, red).

The RPD resides in the central area of DnaG. It covers approximately half of the protein’s volume and contains the protein’s catalytic core (Figure 2B, grey). The N-terminal part of the RPD consists of four β strands (including motifs 2 and 3) and the Toprim-fold (including motifs 4 to 6) [28]. The Toprim-fold is a regulatory portion that constitutes the side wall in the main cleft. This portion binds regulators, metabolites, and metal ions. A phosphotransfer activity, which is usually found among DNA polymerases and topoisomerases, is mediated by two divalent metal ions at the Toprim domain. These two divalent metal ions (mainly magnesium) that bind in close proximity to each other [29] have a crucial role in binding DNA and NTPs to DnaG [30,31]. Further studies have shown that the catalytic core of DnaG may inhabit up to three divalent cations that may vary from Mg^2+^ to Mn^2+^ and Fe^2+^ [32,33].

The ZBD and RPD are both responsible for DNA template binding and primer synthesis. Crystal structures of DnaG-RPD of *M. tuberculosis* [34] and *B. subtilis* [35] in complex with DNA shed some light on the protein-DNA binding interface. However, full characterization of such protein-DNA interactions was elusive since both structures were lacking the ZBD. 

The C-terminal domain (HBD, Figure 2B, orange)) is the least conserved part of DnaG among bacterial species and does not show distinguished features [5]. Interaction of DnaB is mediated by HBD, which, in turn, stimulates the activity of DnaG [36,37]. Despite the variance in the sequence and the structure of DnaG among bacterial species, the HBD consists of two subdomains known as the C1 subdomain, which has a helical bundle, and the C2 subdomain, which has a helical hairpin. In some cases, the tertiary structures of the DnaG-HBD and the N-terminal domain of DnaB of the same species are highly similar, which enables them to bind complementary [37,38]. DnaG-HBD was characterized using x-ray crystallography and nuclear magnetic resonance (NMR). Structures of HBD are available for *B. stearothermophilus* PDB ID: 1Z8S [39], *Helicobacter pylori* PDB ID: 4EHS [40], *Escherichia coli* PDB IDs: 2HAJ, 1T3W [38,41], respectively, *S. aureus* PDB ID: 2LZN [42], and *Vibrio cholera* PDB ID: 4IM9 (Abdul Rehman, S.A. et al., unpublished data). The crystal structure of the complex between the C-terminal part of DnaG (HBD) and the N-terminal part of DnaB from *B. stearothermophilus* sheds light on the binding interface of these two proteins [43].

Emerging evidence for the oligomeric state of DnaG strongly suggest that the active state of primases constitutes higher oligomeric structures ranging from dimers to hexamers [44]. Therefore, if oligomerization of DnaG is physiologically important, then the disruption of the formation of the DnaG oligomer may provide a new drug target.

Based on their amino acid sequence, DnaG primases belong to the Toprim superfamily (which also contains enzymes such as topoisomerases II and IA responsible for altering DNA topology, overcomes the lysogenization defect (OLD) in nucleases, and RecR proteins) [28]. DnaG is also part of the zinc finger CHC2 superfamily that shows high similarity to the ZBD. 

## 4. Challenges in Targeting DnaG Primase

Several reasons may contribute to the challenge of developing DnaG primase inhibitors. Kuron et al. have evaluated DnaG from bacterial genus such as mycobacteria as a drug target for antibiotics [45]. They concluded that, in order to become useful antibiotic agents, inhibitors of DnaG must be highly efficient, i.e., having low K_D_ and high K_i_, that would allow a sufficient decrease of RNA primer formation. 

Another possible reason that contributes to the challenge of finding DNA primase inhibitors resides in the catalytic features of the enzyme. DnaG primase from *Escherichia coli* catalyzes the synthesis of thousands of RNA primers during DNA replication [46]. The RNA primers are formed with a catalytic turn-over rate of hundreds of milliseconds for each nucleotide incorporation event. The rate-determining step in the catalytic activity of DNA primase is the formation of the first phosphodiester bond. It has been suggested that DNA primase acts as a molecular break during DNA replication to keep both polymerases (at the leading strand and the lagging strand) coordinated on the same replisome [47]. The slowness of the catalytic reaction makes primase a difficult drug target since most of the methods used for screening require a sufficient readout. To overcome this challenge, Tosodikov and co-workers have presented a colorimetric assay by using a pyrophosphatase in the reaction mixture as an elegant way of enhancing the readout of primase to a readable threshold [48]. They found a few compounds that can specifically inhibit DNA primase known as suramin, doxorubicin, and ellagic acid (these molecules are also known to have antineoplastic effect), where only doxorubicin was shown to inhibit growth of Mycobacteria [45].

## 5. Potential Types of Inhibition of DNA Primase

**Figure 4:**
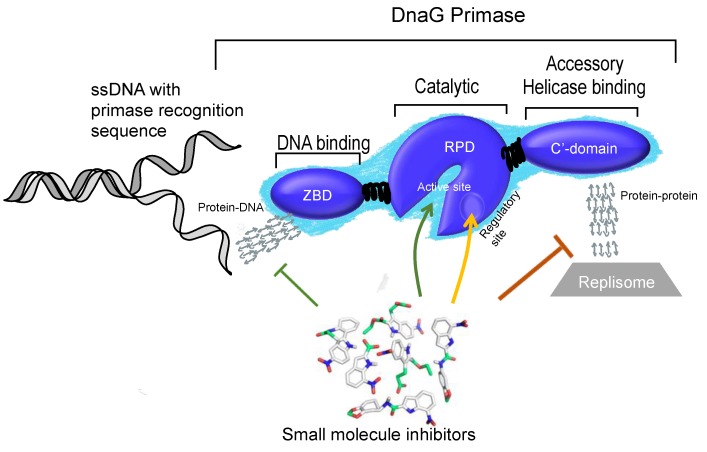
Possible strategies to halt DNA replication through the inhibition of DNA primase: (1) by inhibiting catalytic activity of RNA primers formation; (2) by disrupting the essential protein-protein interactions primase has with other components of the replisome or outside the replisome; (3) by allosteric regulation; and (4) by the prevention of specific DNA recognition.

Inhibition of DNA replication by targeting DNA primase can be obtained by different approaches (Figure 4). These approaches may include direct inhibition of the catalytic activity of RNA primer synthesis, for example, by using antimetabolites that structurally resemble natural substrates and compete with them for binding to the enzyme. Classical competitive inhibitors that act as antimetabolites include ribonucleoside-triphosphate analogues that are used in the clinic as anti-viral and anti-cancer drugs. Due to a high structural similarity with natural nucleotides, the replication enzymes recognize them as substrates for the synthesis of nucleic acids, which leads to the formation of defective DNA or RNA. Inhibition can also be induced by the presence of a chain terminator (nucleotide analogue without 3’ hydroxyl group), as seen in the case of azidothymidine, a reverse transcriptase inhibitor, which is used for the treatment of an HIV infection [49,50]. Additionally, indirect inhibition by non-nucleoside inhibitors that bind to an allosteric pocket causing a conformational change in the enzyme’s active site is possible (e.g., lipiarmycin which affects RNA polymerase and nevirapine which targets reverse transcriptase [51]). Another type of inhibitors cause steric hindrance near the enzyme’s active site include the antibiotic rifampicin. Rifampicin inhibits RNA polymerase by physically blocking the elongation and as a result halt bacterial infection [52]. In addition, approaches for inhibiting DNA replication include the disruption of primase-DNA interactions. Selective interactions between the DNA recognition sequence and the primase is unique and can become a novel target for inhibition (Section 6.4). DNA-binding small molecules have already been used to inhibit transcription factor–DNA interactions, which enables the successful control of gene expression [53]. Similarly, this novel approach can be used to control DNA replication by selectively impeding primase-DNA interactions at the Okazaki fragment start sites (primase recognition sites). Another approach includes the disruption of protein-protein interactions that convey signals to the primosome, from the primosome, and within the primosome (Section 6). 

As described in Section 3, bacterial DnaG contains three functional domains that include different binding sites. Small molecule inhibitors may target the protein in various locations whether they are interaction surfaces with other proteins or specific clefts that binds small metabolites.

**Figure 5:**
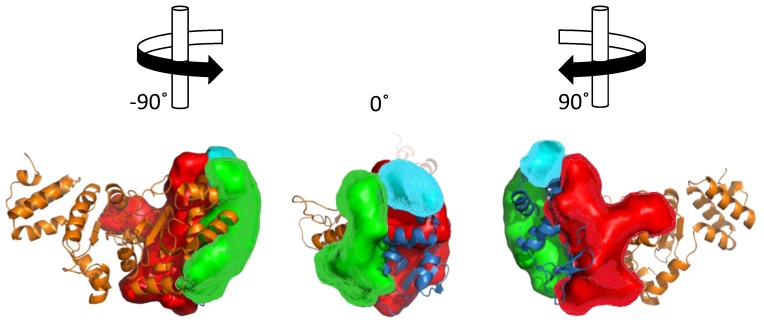
Cleft analysis of DnaG primase. Cleft analysis was performed using the web interface PDBsum (http://www.ebi.ac.uk/pdbsum) and crystal structure of DnaG from *A. aeolicus* (PDB ID 2AU3) [27].

Structural analysis of the ZBD-RPD segment of A. aeolicus (PDB IB 2AU3, [27]) using PDBsum webtool (http://www.ebi.ac.uk/pdbsum) reveals notable clefts in the area next to the zinc finger motif and in the connective area between ZBD and RPD (Figure 5). Most of the extended grooves are related to ZBD and RPD interaction. Two of the substantial clefts are presented in the interspace between the ZBD and the RPD. The major cleft (Figure 5, red) shows a groove extended from the interaction surface and into the catalytic site of RPD. The depth of the main cleft from the surface of RPD is 15.8 Å on average. There are 14.7 buried vertices versus 67.3 accessible ones on average. The second cleft extends from the ZBD-RPD interaction surface and into the ZBD up to the Zn^2+^ binding loop (Figure 5, blue). It has a high amount of positively charged residues that may mediate DNA binding and are probably required to stabilize this interaction. The combined volume of the cleft areas is 8924 Å^3^. The cleft is very flexible and dynamic due to a connective loop between the domains, which means it may be involved or excluded depending on the protein activity and other interactions. Two additional smaller clefts were detected and marked in Figure 5 in cyan and green. They show abundance of aliphatic, aromatic, and non-charged residues that establish the hydrophobic regulatory sites on the RPD.

The ZBD itself is small and planar. Therefore, it does not contain a cleft. Interaction between the two domains (RPD and ZBD), mediated by negative and positive residues on the binding interface, is attributed to a more activated compact structure of DnaG [27]. Small molecules that change the electrostatic pattern of the binding interface and prevent compaction of the enzyme have the potential to become inhibitors. 

The HBD is built from a set of several helices and does not possess significant clefts since most of the amino acids are exposed to the solvent. However, small molecules that disrupt the interaction between DnaG-HBD and DnaB may be useful inhibitors for bacterial DNA replication (Section 6.1).

## 6. Primase Interactions: An Opportunity to Disrupt Essential Activities at the DNA Replication Fork

**Figure 6:**
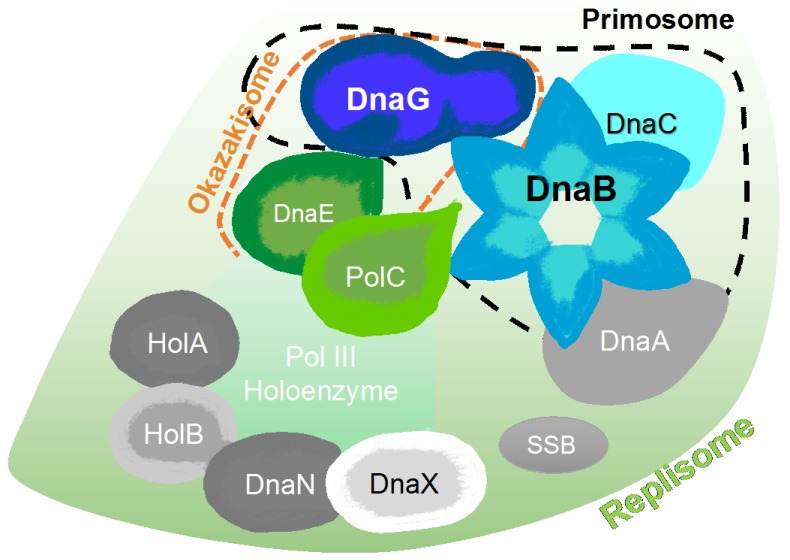
Schematic representation of major protein-protein interactions at a bacterial DNA replication fork created, according to the interaction map of DNA replication proteins [54]. The map is comprised of the DnaA replication initiator, the DnaB helicase, the DnaC helicase loader, the DnaG primase, PolC and DnaE polymerases, the single-stranded DNA binding protein (SSB), the DnaN β clamp, and three clamp loader subunits (HolA, HolB, and DnaX).

Higher order binding organization of DNA primase enables control of its activity. Disrupting some of the interactions of DnaG primase at the DNA replication fork (Figure 6) may provide a useful strategy for drug discovery. 

### 6.1. Primase-Helicase Interactions

DnaB helicase and DnaG primase are two key enzymes in the bacterial replisome that usually work together (in viral systems, the activity of the two enzymes are found in a single polypeptide chain) [55]. Formation of helicase-primase complex results in enhanced priming activity (increased synthesis of RNA primers) [55] and helicase processivity (increased NTPase and DNA unwinding activity) [56]. The interactions between the two enzymes set the replication fork clock [57]. The strength of DnaB-DnaG interactions varies among species. In *E. coli*, this interaction is weak [38,58,59], while it is much stronger in *B. stearothermophilus* (Bst) [56]. However, biochemical properties of DnaB and DnaG in *E. coli* and *B. stearothermophilus* are comparable regardless of these differences in their binding affinities [60]. The C-terminal domain of DnaG primase is required for functional interactions with DnaB helicase [37,61] and it is sufficient to stimulate DnaB activity [56,57]. DnaB helicase forms a hexamer of six identical subunits and operates on the DNA replication fork to separate the double-stranded DNA (dsDNA) into two strands using NTP hydrolysis. In *M. tuberculosis*, for example, the crystal structure of the N-domain of the DnaB helicase forms a hexameric ring [62]. This structure provided the surface for the interaction between the subunits of the hexameric ring and also shed light on the interface available on the helicase for the interaction with the DnaG primase. However, the stoichiometry of DnaG-DnaB binding has not been fully elucidated to date. Bailey et al. crystalized a hexameric form of Bst DnaB as well as DnaB in complex with the C-terminal domain of DnaG [43]. They have obsrved three molecules of DnaG C-terminal domain that bind to the N-terminal collar of DnaB hexamer. Even though this is in accordance with previous studies [56,58], the question remains if only one or two DnaG molecules may be sufficient for priming. Additional information on how these two enzymes work together could help in designing new ways to disrupt their interactions, which could lead to therapeutics.

### 6.2. Primase-SSB Interactions

After the helicase unwinds the dsDNA, re-annealing is prevented by coating the strands with single-stranded DNA binding (SSB) proteins, which are emerging as key components in coordinating replication fork reactions [57,63]. Generally, an unprotected single-stranded DNA (ssDNA) is prone to degradation and recombination [64]. After the helicase unwinds dsDNA, SSB coats the unwound DNA strands and other proteins such as DNA primase. Many more elements are activated [65]. 

The DnaG-SSB interaction is mediated by the C-terminal domain of DnaG and a highly conserved C-terminal hexapeptide motif (DDDIPF) of SSB (SSB-Ct) [66,67]. The binding pocket for SSB-Ct on the C-terminal domain of *E. coli* DnaG has been determined by NMR and it comprises several basic amino acid residues (K447, R452, K518, T450, M451, I455, L519) [68]. Since the SSB-Ct binding pocket is present in other proteins besides DnaG and often mediates interactions that are necessary for bacterial survival, it represents an excellent target for new antibacterial agents [66]. Chilingaryan et al. have combined the saturation-transfer difference NMR (STD-NMR) and surface-plasmon resonance (SPR) to select small molecules that disrupt DnaG/SSB-Ct interaction. The molecules they found presented the simultaneous inhibition of other protein/SSB interactions due to the similar features of the SSB-Ct binding pocket in different proteins [69].

### 6.3. Primase-Polymerase Interactions

In order for coordinated DNA synthesis to take place, primase must initiate and elongate RNA primers and subsequently transfer them to DNA polymerase. The primase may also be involved in leading strand synthesis such as during polymerase stalling. When this event takes place in *E. coli*, DnaG can synthesize the primers on the leading strand to compensate for the stalled DNA polymerase [70]. It is believed that the polymerase can then be transferred from the blocked site to the RNA primer site based on the activity of clamp-clamp loader. 

In eukaryotes, there is tight interaction between DNA primase and polymerase, which enables the direct transfers of oligoribonucleotides from primase to polymerase [71]. In contrast, the interactions between primases and polymerases in prokaryotic systems are more transient, which means that the primase can associate and dissociate from the replication fork in order to catalyze several rounds of primer synthesis [37,72,73].

It has been shown in *E. coli* that DNA polymerase has the ability to restrict the length of oligoribonucleotides synthesized by the primase [74,75]. The proposed mechanism involves displacement of DnaG primase by one of the PolIII subunits belonging to the clamp-loader complex [76]. In *B. subtilis*, two DNA polymerases are active in the DNA replication fork including PolC, which is a high-fidelity polymerase that acts on both DNA strands, and DnaE_Bs_, which is a low-processive DNA polymerase involved only in the lagging strand synthesis [77,78,79]. The initiation and elongation of RNA primers is carried out by a protein complex comprised of DnaG, DnaE_Bs_, and replicative helicase (DnaC). The DnaG synthesizes primers extended by DnaE_Bs_ and transferred to PolC [80].

### 6.4. DNA-Primase Interactions at the Okazaki Fragment Start Sites: A Novel Drug Target

Specific DNA-protein recognition includes selective binding of proteins to a particular DNA sequence, which is important for many cellular processes. This includes DNA replication, repair, and recombination [81]. Of all the protein-nucleic acid interactions at the DNA replication fork, primase binding to DNA is specific and unique. DNA-dependent RNA primer synthesis by DnaG-type primases involves the recognition of a tri-nucleotide DNA sequence and is followed by the synthesis of a dinucleotide, which is then extended into a functional primer by a DNA primase [82,83]. The trinucleotide recognition sequence of the template DNA consists of a cryptic nucleotide at the 3′-end of the DNA template, which is recognized by the primase but is not copied into the synthesized primer [84]. 

The N-terminal Zinc binding domain of DnaG mediates the specific DNA sequence recognition. Despite extensive research to date on DNA primases, the precise role of the zinc motif in sequence recognition and primer synthesis remains to be resolved. In the absence of the ZBD or DNA [85], extensively studied DNA primase from *bacteriophage T7* synthesizes random di-ribonucleotides in a DNA-independent manner. 

Even though a specific trinucleotide sequence is recognized by DNA primase, flexibility in selecting initiation sites for Okazaki fragments is allowed [86], which means not every primase recognition site will become an Okazaki fragment start site. Additional information regarding such DNA binding is required to better understand the specific interaction between DnaG and DNA.

Recent advances in technological tools allow analyses of specific binding preferences of DNA primase [87]. High throughput techniques have yielded data on genomic binding specificities that can be used to determine factors that govern the binding of DnaG primase to Okazaki fragment start sites. As mentioned above, specific DNA sequence recognition is performed by the zinc binding domain of DnaG primase. Disruption of such specific binding is likely to hamper the formation of Okazaki fragments and, as a result, halt DNA replication. It is, therefore, assumed that such inhibitors would be very specific and unique. Developing ways to control the specific recognition by DnaG primase can yield a new-class of inhibitors.

## 7. The Development of Novel Therapeutic Approaches

Antibiotic-resistant bacterial strains emerged almost immediately after the introduction of penicillin [88]. Since then, an overuse of antibiotics has led to increased drug resistance and several pathogens have developed multi-drug or pan-drug resistance [89,90]. Even though many new antibiotics have been developed in recent decades, once a drug is used clinically, resistance will eventually develop. Therefore, new antibacterial compounds are in constant demand. In contrast, the current pipeline in the search for new antibacterial agents is expanding slowly and is inadequate for the development of a completely novel regimen. Most of the available antibacterial drugs do not follow Lipinski’s rule of 5, which evaluates the “drug-likeness" of a chemical compound based on its physico-chemical properties [91].

Target selection is one of the most rate-limiting steps in the discovery of every new antibiotic [92] since it requires that: (a) the target gene product is essential and inhibition of its function kills or inhibits the growth of the bacteria; (b) the target is structurally different from mammalian proteins (avoiding mechanism-based toxicity); (c) the target is “druggable”, i.e., has a reasonable site to which small molecules can bind and exert a biological effect; (d) the target possibly has low propensity for a rapid development of resistance [92]; and (e) the structure of the target is conserved across bacterial species to provide a broad antibacterial spectrum. After identifying a suitable drug target, a search for a starting compound begins. Once it is available, additional optimization cycles into a clinical candidate are required to improve potency through a pharmaceutical development plan. Often, academic institutions can initiate the drug development process and performs the lead discovery phase after which the pre-clinical/clinical development takes place mostly in pharmaceutical companies [93]. The purpose of the “academic stage” is, therefore, to identify the relevant compounds and to provide useful information (through the investigation of many structure-activity relationships) that will be used to optimize a compound into a potent lead. 

Proteins that reconstitute the bacterial DNA replisome including DnaG primase fulfills all the above-mentioned criteria and can potentially serve as attractive targets for drug discovery. 

## 8. Approaches in Screening for DNA Primase Inhibitors

Different approaches were used for the initial screening process of small molecules that bind/inhibit DNA primase: high throughput screening (HTS), fragment-based screening, and virtual screening [94]. Koupsell et al. reported a non-radioactive primary assay for the use of HTS [95]. This assay relies on the formation of a stable DNA-RNA primer hybrid that can be detected by pico-green. Although a fluorimetric assay yielded hits, the requirements for larger RNA primer and lack of sensitivity in the detection could indicate non-specific binding. Afterward, Tsodikov and coworkers developed a method to detect the amount of pyrophosphate released in every incorporation of nucleoside mono-phosphate to the elongated RNA primer by DnaG from *M. tuberculosis* [48]. This assay couples the use of inorganic pyrophosphatase to break-down pyrophosphate molecules into two inorganic phosphate molecules. In this way, the signal was amplified so that the colorimetric assay could be adapted for the purpose of HTS [48]. 

In antibacterial research, HTS has not enriched the drug development pipeline with new lead compounds [96,97]. Typically, HTS cannot indicate the mechanism of the action of the drug and its results are often ambiguous and misleading (e.g., false positives obtained in an HTS can be very costly and hit rates vary between 0.1% and 2%) [98]. Overall, the low rate of identifying new therapeutic agents by using the standard HTS paradigm has been associated with the relatively limited repertoire of the libraries that were used [97]. 

As mentioned earlier, the slow catalytic rate of primase is expected to yield a very low readout and, therefore, the most common way of tracking primase activity biochemically is by measuring the incorporation of radiolabeled nucleotides to the newly synthesized RNA primer. 

While HTS could filter-out positive hits, fragment-based screening has emerged as a major approach of hit to lead discovery in the research of infectious diseases [99] where conventional approaches in drug discovery have failed. Unlike HTS, fragment-based screening monitors the binding of small molecules rather than the readout of the target’s biochemical process [100]. The detection of binding of such fragments is often very weak and, therefore, biophysical methods (such as NMR, SPR, calorimetry, or X-ray crystallography) are used to monitor the binding [101]. Despite the traditional belief that low affinity and low weight hits would not build into a clinical candidate, molecules found by using fragment-based screening are emerging in the late stages of clinical trials [100]. The use of fragment molecules for screening is advantageous over the molecules in traditional HTS libraries, which are larger and more lipophilic. Molecules in HTS libraries fulfill Lipinsky’s “rule of five” [102], which occasionally enforces researchers to compromise on the disposition properties to obtain potent inhibitors. However, the small-size molecules in fragment libraries increased the chances of binding through weak interactions. The generation of hits by screening fragment molecules with NMR as the main tool in structure-activity relationship was introduced in the 1990s [103] and was followed by the conceptual use of fragment libraries [104,105] that included diverse compounds with high solubility. For a review on detection methods and the type of libraries, see Reference [94]. Lastly, virtual screening is a computational approach that simulates the binding properties of virtual compounds and calculates the efficiency of interactions based on known physical principles. Usually, there is a need for an atomic resolution structure of the target macromolecules (e.g., NMR or crystal structure). However, the success rate of virtual screening is low and, therefore, it is not a stand-alone strategy but is used to complement other drug discovery approaches [93].

## 9. Molecules that were Found to Inhibit DNA Primase 

**Figure 7:**
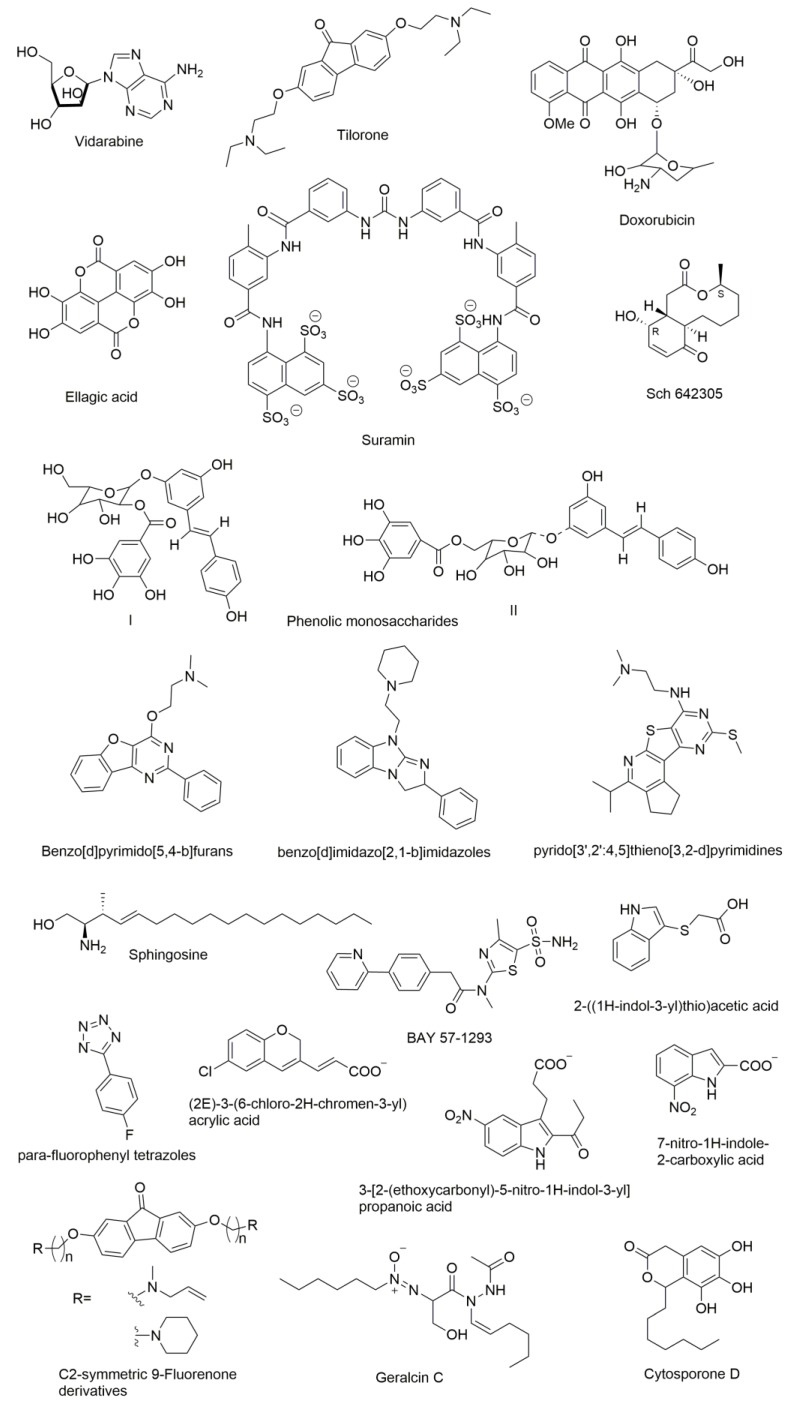
Chemical structures of DnaG primase inhibitors.

Several compounds have been reported as DnaG inhibitors that can be potentially used as antimicrobial agents (molecular structures of the compounds are presented in Figure 7). Regardless of their highly therapeutic potential, none of them have emerged as clinical candidates [10].

DnaG primase inhibitors can be categorized into two classes: NTP analogues and non-NTP analogues. Most of the compounds known to inhibit primases are nucleotide analogues such as AraATP (Vidarabine) and 2-fluoro-AraATP. NTP analogues containing arabinofuranosyl sugars inhibit both eukaryotic and herpes virus primase [106,107]. The AraATP is the active form of vidarabine and can be used both as an inhibitor and a substrate for the primase. For example, *E. coli* DnaG may utilize 2′,3′-dideoxynucleoside 5′-triphosphates (ddNTPs) as substrates. Once they are incorporated, elongation can no longer occur due to the lack of a 3′ hydroxyl that disables a formation of phosphodiester bond with the next nucleotide [108]. However, there are several known inhibitors of DnaG that are non-NTP analogues. Biswas et al., recognized three compounds that inhibit *M. tuberculosis* DnaG and are not NTP analogues [109]. They reported a non-radioactive primase-pyrophosphatase activity assay for screening primase inhibitors. HTS was then used and it identified suramin, doxorubicin, and ellagic acid as compounds with primase inhibition activity.

Both suramin and doxorubicin contain aromatic structures with polar functional groups and are potent (low-mM) DNA and nucleotide triphosphate competitive inhibitors. These molecules are likely to interact with more than one site on *M. tuberculosis* DnaG in order to block DNA and/or NTP binding. Owing to its polyanionic character, suramin may interact with some of the same sites on DnaG that contact phosphate groups of the DNA backbone or incoming NTP. Furthermore, the amide bond present in suramin might be essential for its selectivity because of favorable hydrogen bonding interactions. Suramin is also known to inhibit eukaryotic DNA primase by competing with GTP and it likely inhibits DnaG via a similar mechanism [108]. In contrast, another DnaG inhibitor known as tilorone, which is a 9-fluorenone based compound, is more potent in low-micromolar concentrations against *B. anthracis* than *M. tuberculosis* DnaG despite homology between these enzymes, which suggests that DnaG can be targeted selectively [109]. Other C2-symmetric fluorenone derivatives with a long carbon chain were shown to inhibit growth of *B. anthracis*, *S. aureus*, *Methicillin-resistant Staphylococcus aureus* (*MRSA*), *F. tularensis*, and *B. thailandensis*, which suggests that these molecules may also have a broad-spectrum of antimicrobial activity [110].

Apart from the above categorized inhibitors, there are several other inhibitors reported in the literature that showed different mechanisms. Bicyclic 10-membered macrolide Sch 642305 is a novel primase inhibitor isolated from the *Penicillium verrucosum* that exhibits inhibitory activity against bacterial DNA primase with an EC_50_ of 70 µM [111]. Phenolic monosaccharides (I & II) obtained from the methanolic extract of the Peruvian plant *Polygonum cuspidatum* were identified as inhibitors of the bacterial DNA primase enzyme with an IC_50_ of 4 µM and 5 µM, respectively [112]. Other natural products known as cytosporone D [113] and geralcin C [114] were shown to inhibit DnaG primase of *E. coli* with an IC_50_ of 250 µM [115] and 700 µM, respectively.

Agarwal et al. described the lead primase inhibitors and a 3D pharmacophore for the primase inhibition activity. Benzo[d]pyrimido[5,4-b]furans [116], benzo[d]imidazo[2,1-b]imidazoles [10], and pyrido[3′,2′:4,5]thieno[3,2-d]pyrimidines [116] are the lead inhibitors of *E. coli* DnaG Primase. Three-dimensional pharmacophore development suggested inhibitors that contain two hydrophobes (H), two hydrogen bond acceptors (A), and a donor (D) group. Furthermore, an aromatic ring fused with furan, pyrimidine, or imidazole imparts satisfactory primase inhibition. Several nitrogen-bearing functional groups such as piperazine, morpholine, and aliphatic amines attached to quinazoline analogs also enhance the activity [116]. Due to their very different chemical structures, these inhibitors seemingly act by unrelated mechanisms. For example, the phenolic monosaccharides were shown to inhibit the binding of primase to ssDNA [112] while furans, imidazoles, and pyrimidine derivatives bind to the RNA Polymerase Domain (RPD) of primase to establish inhibition [116]. The mechanism of inhibition has not yet been determined for the latter set of compounds.

There is another class of compounds that are potent inhibitors of mammalian DNA primase in vitro [117]. Sphingosine, phytosphingosine, and N, N-dimethylsphingosine strongly inhibit the activity of purified calf thymus DNA primase and also inhibit the growth of the human leukemic cell line HL-60, which exerts strong cytotoxicity. Dihydrosphingosine and cis-sphingosine that moderately inhibit cell growth *in vivo* but show indirect inhibition of DNA primase in vitro cause cell death [118]. Kleymann et al. reported new helicase-primase inhibitors as drug candidates for the treatment of herpes simplex disease. BAY 57-1293 (*N*-[5-(aminosulfonyl)-4-methyl-1,3-thiazol-2-yl]-*N*-methyl-2-[4-(2-pyridinyl)phenyl]acetamide) is the new inhibitor of the HSV helicase-primase with potent in vitro anti-herpes activity with a novel mechanism of action [119]. Ilic et al. identified small molecule inhibitors of the T7 DNA primase by using fragment-based screening by NMR and virtual filtration. Three small molecule inhibitors (2E)-3-(6-chloro-2*H*-chromen-3-yl) acrylic acid, 3-[2-(ethoxycarbonyl)-5-nitro-1*H*-indol-3-yl]propanoic acid, and 7-nitro-1*H*-indole-2-carboxylic acid were reported. All three of these molecules were shown to bind to the active site of primase and are expected to interfere with the binding of substrate (ribonucleotides) or the DNA template [120]. Recently, Chilingaryan et al. identified inhibitors of the primase/SSB-Ct interaction using fragment-based screening by a saturation-transfer difference nuclear magnetic resonance (STD-NMR) and surface plasmon resonance assays [69]. Compounds containing indole, 2-((1*H*-indol-3-yl)thio)acetic acid and 1H-tetrazole scaffold especially para-fluorophenyl tetrazoles were identified as first-generation hits. These compounds showed various electrostatic and hydrogen-bond networks within the binding pockets, which makes them promising starting points for further optimization [69].

Some of the compounds are specific inhibitors of the primase of Gram-negative bacteria while others show a broader range of inhibition [121]. Factors to consider in the discovery and optimization of inhibitors are molecular diversity and the drug-like characteristics of compounds such as H-bond donors, H-bond acceptors, aromatic centers, salt bridge formation, and pharmacokinetics (adsorption, efflux, metabolism, excretion) [121]. Compounds bearing additional polar groups such as N(CH_3_)_2_, morpholino, or OH groups attached through a linker to the aromatic core exhibit higher potency. These findings suggest that the polar groups make additional favorable interactions with the primase enzymes [116]. In general, SAR reveals that compounds with bulky substituents are inactive while smaller substituents such as OH, CH_3_, SCH_3,_ N(CH_3_)_2,_ COOCH_3_, OCH_3_, and OCH_2_CH_3_ are tolerated. Therefore, further optimization of the aromatic-based primase inhibitors may provide novel small molecules for anti-bacterial therapy.

## 10. Conclusions

Even though DnaG is an excellent drug target, it was postulated that almost total inhibition is required to affect bacterial cell viability. The requirements to make DnaG effective drug targets are: (1) combinations with other antibacterial drugs to maximize the effect when other treatments fail; (2) maintaining high intracellular concentrations of DnaG inhibitor by co-inhibition of other mechanisms such as efflux pumps or enzymatic degradation of the inhibitor; (3) improved drug-delivery systems to direct large amount of inhibitors into bacterial cells; (4) designing inhibitors that will prevent the development of bacterial resistance by targeting essential amino-acids; and (5) development of noncompetitive irreversible inhibitors to increase the effective concentration required to impair DnaG primase and halt DNA replication.
